# Long-term monitoring of a full-scale parallel-unit downflow hanging sponge system with adaptive capacity for fluctuating wastewater flows in Japan

**DOI:** 10.1007/s11356-026-38300-y

**Published:** 2026-10-13

**Authors:** Yasuyuki Takemura, Shinichi Yamazaki, Takashi Matsueda, Satoshi Ohmori, Akihiro Nagano, Motofumi Taniwaki, Noriko Tomioka, Masataka Aoki, Kazuaki Syutsubo

**Affiliations:** 1 Regional Environment Conservation Division, National Institute for Environmental Studies (NIES), 16-2 Onogawa, Tsukuba, Ibaraki 305-8506 Japan; 2 Department of Civil Engineering and Architecture, National Institute of Technology (KOSEN), Maizuru College, 234 Shiroya, Maizuru, Kyoto, 625-8511 Japan; 3 Sanki Engineering Co., Ltd., 7-10-1 Chuorinkan, P.C., Yamato, Kanagawa 242-0007 Japan; 4 Water and Sewerage Division, Susaki City Hall, 2-3-11 Nishimachi, Susaki, Kochi, 785-0009 Japan; 5 Research Center for Water Environment Technology, School of Engineering, The University of Tokyo, 7-3-1 Hongo, Bunkyo-Ku, Tokyo, 113-8656 Japan

**Keywords:** Downflow hanging sponge, Parallel-unit design, Decentralized wastewater treatment, Energy efficiency, Sludge reduction, Flow adaptability

## Abstract

This study presents a 30-month full-scale evaluation of a parallel-unit downflow hanging sponge (DHS) system with adaptive downsizing capability at the Susaki Sewage Treatment Plant, Japan. The DHS reactor comprised ten identical units operated in parallel, allowing capacity adjustment by varying the number of active units. The system (primary sedimentation tank → DHS → moving bed biological filter) was monitored under normal, low-temperature (winter), partial downsizing, and sponge sheet installation (upper part of the sponge media-filled segment) conditions. Dichromate-based chemical oxygen demand (COD_Cr_), biochemical oxygen demand (BOD), total suspended solids (TSS), NH₄⁺–N, NO₃⁻–N, total nitrogen, and *Escherichia coli* were analyzed weekly and monthly to evaluate organic removal, nitrification, and microbial reduction. DHS effluent (77–85% COD_Cr_, BOD removal, and a 1.5 log₁₀ reduction in *E. coli*) consistently met Japanese discharge standards. Downsizing to nine units, that is, reducing the hydraulic retention time, maintained effluent quality, confirming the system’s flexibility under declining inflows. Statistical comparison among operational periods showed no significant difference in NH₄⁺–N loading rate, whereas NH₄⁺–N removal efficiency was significantly higher after sponge sheet installation. The sponge sheet enhanced hydraulic dispersion and nitrification, reducing NH₄⁺–N from 16–19 to 9 mg N·L⁻^1^ and promoting NO₃⁻–N accumulation. Compared to the conventional activated sludge process previously employed, DHS used 83% less power and reduced excess sludge generation, demonstrating its potential as a sustainable, energy-efficient alternative for small- to medium-scale sewage treatment facilities with aging infrastructure. These findings highlight DHS as a resilient and socially viable solution for regions with declining populations, ensuring reliable wastewater treatment despite demographic and economic challenges.

## Introduction

Decentralized wastewater treatment systems are considered viable and necessary alternatives for wastewater management in peri-urban and rural areas (Bernal et al. 2021). These systems are expected to be low-cost, flexible in design, and highly adaptable, with demand particularly high in developing countries (Ventura et al. 2024). In Japan, where centralized sewerage infrastructure is well established, innovative decentralized wastewater treatment systems have recently attracted attention due to population decline and aging facilities in regional cities.

Modern sewerage treatment in Japan began in 1922 with the introduction of trickling filter systems (Ichikawa 1988). As of 2025, the nationwide sewage treatment population coverage ratio has reached 93.7% (Ministry of Land, Infrastructure, Transport and Tourism 2025). Among the 2457 municipal sewage treatment plants (STPs) registered with the Japan Sewage Works Association, large-scale facilities serving more than 10,000 individuals accounted for 41.6%, while medium-scale facilities serving fewer than 2000 individuals accounted for 21.0%. The treatment processes include activated sludge methods (33.1%), oxidation ditches (40.5%), advanced treatment processes, such as the anaerobic–oxic process (20.8%), and membrane bioreactors (5.5%). Advanced treatment processes are common in large urban facilities, whereas small-scale facilities typically use activated sludge or oxidation ditch systems (Japan Sewage Works Association 2018).

In addition, Japan has 5353 rural community wastewater treatment facilities, 79.7% of which serve 200–2000 individuals. These facilities often employ modified activated sludge or membrane bioreactor processes (Japanese Agricultural Village Drainage Data Files 2020). Approximately 30% of these facilities have been operating for more than 30 years, highlighting the need for countermeasures against aging and facility renewal. Furthermore, population decline is expected to reduce sewage inflow and the associated revenue from sewerage fees, posing significant challenges. Nationwide, sewage treatment volumes have been decreasing since fiscal year 2015, particularly in regional cities and rural areas (Japan Sewage Works Association 2018).

Conventional activated sludge and membrane bioreactor systems determine treatment capacity by tank volume, making it difficult to reduce costs and energy consumption as inflow decreases. To address this issue, technologies that allow flexible downsizing of treatment capacity and cost reduction are required. In response, the National Institute for Land and Infrastructure Management launched a demonstration study under the B-DASH project in 2017, titled “Demonstration of technology adaptive to flow fluctuation using DHS system.”

The downflow hanging sponge (DHS) process has been widely studied in several countries as a post-treatment system for upflow anaerobic sludge blanket (UASB) reactors. DHS uses a polyurethane sponge as a biomass carrier in a trickling filter configuration and offers several advantages: (i) reduced energy consumption for aeration, (ii) long-term retention of high-concentration biomass with low excess sludge production, and (iii) simple operation and maintenance (Tyagi et al. 2021). In addition to organic matter removal, DHS reactors can promote both nitrification and denitrification because oxygen gradients develop within the sponge media, allowing nitrogen transformation and partial nitrogen removal within the reactor. Moreover, DHS comprises multiple units arranged in parallel, which enables easy downsizing by adjusting the number of active units.

The DHS system evaluated in this study represents the first full-scale installation for direct domestic sewage treatment in Japan. It was introduced in 2017 at the Susaki STP in Kochi Prefecture, replacing the extended-aeration activated sludge process originally designed for 1800 m^3^·d⁻^1^ in 1976. Due to population decline and economic constraints, the new DHS system was designed for 500 m^3^·d⁻^1^. This significant reduction in design capacity reflects the need for flexible, cost-effective treatment solutions, as population decline in the City of Susaki continues and economic constraints make an increase in the sewerage connection rate unlikely. The total height of the DHS system is approximately 5 m, which theoretically allows for a design that utilizes the existing aeration tank structure of a conventional activated sludge process (typically 4–5 m high). However, the old aeration tank deteriorated and failed to meet structural strength standards; therefore, a new DHS system was designed and constructed.

Previous studies on DHS systems have predominantly addressed their use as post‑treatment units following anaerobic reactors or have been limited to pilot‑ and package‑scale applications operated under relatively stable conditions. These studies primarily focused on reactor development and treatment performance in pilot- or full-scale DHS systems operated as post-treatment units following anaerobic reactors (Onodera et al. 2014b; Okubo et al. 2015; Nomoto et al. 2018; Tyagi et al. 2021). However, reports on the full-scale renovation of existing activated sludge facilities to DHS-based systems and their long-term operational performance remain limited, particularly in temperate regions, as most full-scale operational experience has been obtained under tropical or subtropical conditions. Consequently, long‑term performance data from full‑scale DHS systems directly treating domestic sewage under practical operational constraints remain scarce. In particular, the adaptability of parallel‑unit DHS configurations to sustained flow declines and seasonal temperature variations, as well as the hydraulic and biological impacts of simple head‑end modifications, have not been sufficiently elucidated.

To the best of our knowledge, this study provides one of the first long-term evaluations of a full-scale parallel-unit DHS system directly treating domestic sewage in a temperate region. In this study, the long-term treatment performance of the DHS system was evaluated based on key water quality parameters (dichromate-based chemical oxygen demand (COD_Cr_), biochemical oxygen demand (BOD), and suspended solids (SS), NH₄⁺–N, and NO₃⁻–N). Considering that Japan revised its effluent standard in April 2025, replacing total coliforms with *Escherichia coli* as the fecal pollution indicator, the removal efficiency of *E. coli* in the DHS system was also assessed. Additionally, operational aspects, such as power consumption and excess sludge generation, were comparatively evaluated.

Given the projected population decline in the City of Susaki, DHS operations may need to be scaled back in the future. In practice, the key question is whether capacity can be adjusted while maintaining stable performance over extended periods and under variable environmental and operational conditions. Addressing this question requires full‑scale, long‑term verification of operational flexibility rather than short‑term performance tests alone. To examine this feasibility, from April to December 2022, one of the ten DHS units was intermittently shut down in rotation to simulate downsizing, and the stability of treatment performance under this condition was evaluated. In addition, in March 2023, a polyurethane sponge sheet was installed directly below the wastewater distributor at the top of the DHS reactor, above the first sponge-media-filled segment, to improve influent distribution and enhance organic matter removal. Continuous operation has been maintained since installation. Accordingly, this study is positioned at the practical frontier of DHS implementation by combining extended full‑scale monitoring with intentional operational modifications under routine plant operation. We hypothesized that (i) the parallel-unit DHS configuration would maintain stable treatment performance during downsized operation, and (ii) installation of a sponge sheet would improve wastewater distribution and consequently enhance nitrification performance.

## Materials and methods

### DHS reactor operation

**Fig. 1:**
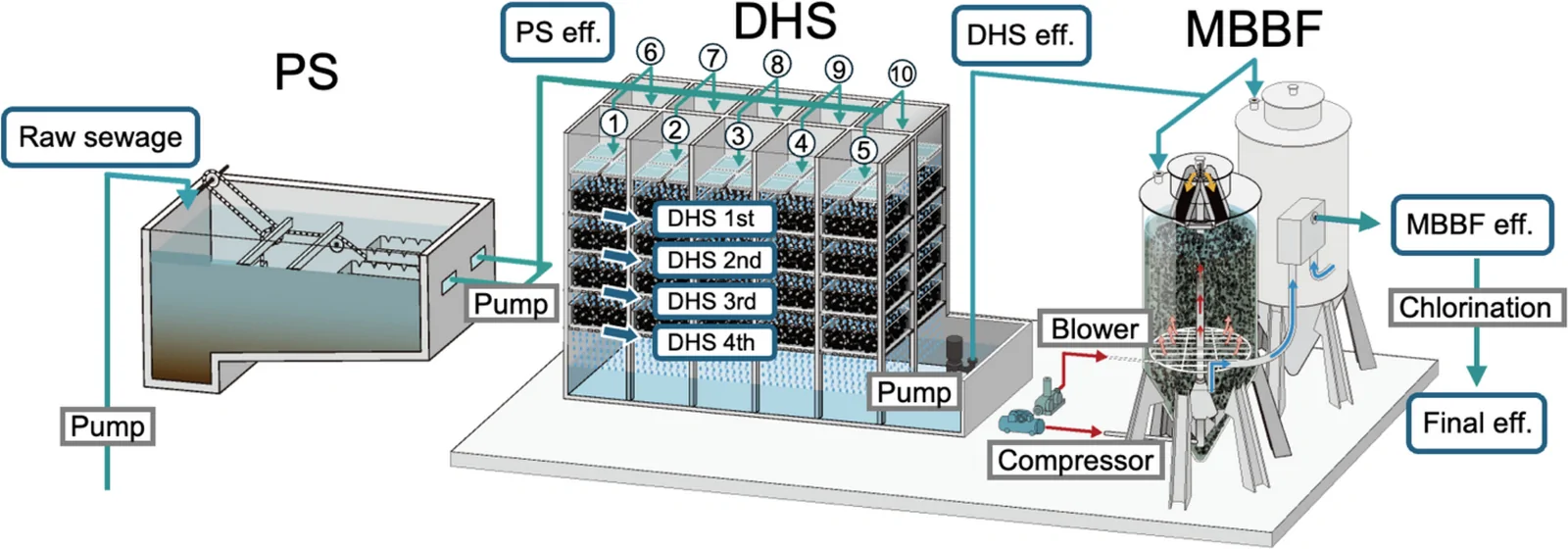
Schematic of the full-scale downflow hanging sponge (DHS) system installed at the Susaki Sewage Treatment Plant (STP). The system consists of ten DHS units followed by a moving bed biological filter (MBBF), designed to allow flexible adjustment of treatment capacity. Circled numbers at the DHS head indicate the corresponding unit numbers. PS, primary sedimentation tank

The full-scale DHS system was operated at Susaki STP (33° 24′ 18″ N, 133° 17′ 19″ E) in Susaki, Kochi, Japan. The design capacity of the STP was 500 m^3^·d⁻^1^, with an average operational flow of 365 m^3^·d⁻^1^. The treatment train comprised a primary sedimentation tank (PS), a DHS reactor, and a moving bed biological filter (MBBF) for polishing (Fig. 1).

For hydraulic comparison, theoretical hydraulic retention times (HRTs) were calculated using the average flow of 365 m^3^·d⁻^1^ during the monitoring period. Raw sewage was pumped from a manhole to an automatic bar screen to remove large solids (> 2.5 mm) and then conveyed to the PS. The PS had a surface area of 9.6 m^2^ and a depth of 3.0 m, yielding a volume of 28.8 m^3^ (HRT ≈ 1.9 h).

The PS effluent was distributed among ten identical DHS units operated in parallel. Each unit consisted of four stacked container segments (2.0 m (length) × 2.0 m (width) × 0.78 m (height) per segment). The total cross-sectional area of the DHS was 40 m^2^, and the cumulative sponge height was 3.12 m. The DHS employed randomly packed polyurethane sponge carriers enclosed in polyethylene cylindrical net frames (33 mm in diameter × 33 mm in length). The total sponge volume was 64.0 m^3^, corresponding to a design theoretical HRT ≈ 4.2 h at 365 m^3^·d⁻^1^.

Downstream of the DHS, the MBBF consisted of two filters (each 6 m^2^ in area × 2.5 m in height) filled with anthracite filter media (4–10 mm). The active water volume was approximately 15 m^3^ (theoretical HRT ≈ 1.0 h), with a porosity of approximately 50%. The MBBF was continuously aerated throughout the study period. The final effluent was disinfected by automatic chlorination using a solid chlorine compound and discharged into the Mitarai River near the coast.

To evaluate the impact of capacity downsizing on treatment performance, the plant was operated for three distinct periods:

1. June 2021–March 2022 (Period 1; normal operation)—all 10 DHS units in service (64.0 m^3^ sponge; theoretical HRT ≈ 4.2 h).
2. April 2022–December 2022 (Period 2; partial downsizing)—9 DHS units intermittently operated in rotation (57.6 m^3^ sponge; theoretical HRT ≈ 3.8 h). During this period, Unit 1 was continuously supplied with PS effluent to assess flow distribution profiles, while the remaining nine units were periodically stopped (at 1-week intervals) to prevent drying and simulate downsizing.
3. March 2023 onward (Period 3; sponge sheet installation)—a polyurethane sponge sheet (≈20 mm thick) was installed directly beneath the wastewater distributor, covering the full cross-sectional area across the 10 DHS units. The plant was then continuously operated to monitor the effects of the sponge sheet on hydraulic distribution and process performance.

### Sampling and analysis

Grab samples of the raw sewage and DHS effluent were collected once weekly between 09:00 and 10:00. Based on our prior observations at the Susaki STP, BOD, NH₄⁺–N, and flow rate during this time window tend to be higher than the daily average, providing a conservative basis for performance evaluation.

The following parameters were analyzed: COD_Cr_, total nitrogen (TN), ammonium (NH₄⁺–N), nitrate (NO₃⁻–N), BOD, total suspended solids (TSS), *E. coli*, and total coliforms.

- COD_Cr_ (Method 8000, HACH), TN (Method 10071, HACH), NH₄⁺–N (Method 10205, HACH), and NO₃⁻–N (Method 10206, HACH) were measured using standard colorimetric methods.
- *E. coli* (blue colonies) and total coliforms (blue and purple colonies) were enumerated using Compact Dry EC plates (Nissui). Microbiological tests were performed within 36 h of sampling after the samples were transported to the laboratory.
- BOD (unfiltered) and TSS were analyzed on-site at the Susaki STP following the Japanese Sewage Works Association (JSWA) Wastewater Examination Method (JSWA 2012).

Weekly measurements were conducted from June 2021 to December 2022. From March 2023 onward (i.e., following installation of the sponge sheet), BOD and TSS continued to be monitored weekly, while NH₄⁺–N and TN in raw sewage and DHS effluent, and NO₃⁻–N in DHS effluent, were measured monthly in accordance with the JSWA Wastewater Examination Method (JSWA 2012). Unless otherwise specified, values are reported as means ± standard deviations. Detection limits and quality control procedures (instrument calibration, reagent blanks, and duplicate analyses) were conducted in accordance with the manufacturer’s instructions and JSWA standard methods.

BOD, COD_Cr_, and NH_4_^+^–N volumetric loading rates were calculated by dividing the daily mass loading by the active sponge volume of the DHS reactor (64.0 m^3^ during normal operation and 57.6 m^3^ during downsized operation).

In addition, dissolved oxygen (DO) and oxidation–reduction potential (ORP) were measured at the first to fourth DHS segments during reactor profiling surveys conducted in August and November 2021 and August and November 2023. DO was measured using a portable optical dissolved oxygen meter (FDO 925; WTW), and ORP was measured using a portable ORP meter (ORP60-Z; Apera Instruments). These measurements were used to evaluate changes in oxygen availability and oxidation conditions before and after the installation of sponge sheets.

Statistical analyses were conducted using R (version 4.6.0) and the rstatix package (version 1.0.0). Normality of the datasets was assessed prior to statistical analyses. Differences among operational periods were evaluated using Welch’s one-way analysis of variance (Welch ANOVA), which does not assume homogeneity of variances. When significant differences were detected, pairwise comparisons were conducted using the Games–Howell post hoc test. Statistical significance was defined as *p* < 0.05.

### Measurement of sludge concentration on sponge carriers

To evaluate the effect of temporary feed stoppage (partial downsizing) on the biomass retained by the sponge carriers, we measured the sponge-attached sludge concentration under controlled laboratory conditions that reproduced the DHS internal environment (humid, dark, and aerated). Briefly, a 200-L opaque polyethylene tank was used as the test chamber. Water was added to a depth of several centimeters, and an air pump was used to maintain ventilation. Sponge carriers were obtained from the first segment of Unit 1 in the full-scale DHS reactor. Using plastic mesh frames (10 cm width × 20 cm height) to mimic a DHS segment, 4 mock DHS segments were prepared, each loaded with 10 sponge carriers, and placed on a stand to prevent contact with water. This setup ensured ventilation and shading while preventing the sponges from submerging, thereby replicating the conditions of the DHS reactor. Experiments were conducted indoors without direct sunlight (room temperature, 13–21 °C).

Sponge carriers were sampled at four time points: immediately after setup (Day 0) and on Days 3, 8, and 14. These sampling intervals were selected to represent short-term (3 days), intermediate (approximately 1 week), and extended (2 weeks) feed-stoppage conditions considered operationally realistic for rotational downsizing management. At each time point, three sponge carriers were collected from each mock segment and placed in zip-type polyethylene bags. While rinsing the support frames inside the bag, they were removed to retain only the sponge material. Then, 100–200 mL of water was added to the bag, and the sponge was repeatedly compressed and released to elute the attached sludge into suspension. This extraction step was repeated at least five times. The resulting suspension was transferred to a 1-L polypropylene container, and the total volume was adjusted to 1 L.

The suspensions were analyzed for SS and volatile suspended solids (VSS). The concentrations were expressed per unit sponge volume (grams of suspended solids or grams of volatile suspended solids (gVSS) per liter of sponge) using the known geometric volume of each sponge carrier. In addition, periodic samples collected directly from the full-scale DHS reactor body were analyzed in the same manner for comparison with the mock-segment results.

To assess how feed stoppage influenced sludge properties, we compared the VSS/SS ratio across the first to fourth segments (DHS 1st–4th) of Units 1, 5, 6, and 9, sampled on May 18, 2022, during the partial downsizing period. Units 1 and 9 had a continuous supply, whereas Units 5 (2 weeks after restarting) and 6 (1 week after restarting) experienced a 1-week feed stoppage followed by restarting. These units were selected to compare continuously operated conditions with units recovering from temporary feed stoppage. Segment-wise VSS/SS profiles were used to infer changes in the biomass fraction associated with temporary feed stoppage and subsequent recovery.

### Tracer test

To determine the actual HRT of the DHS reactor, a tracer test was conducted following the method described by Danshita et al. (2020). Each DHS unit head was equipped with 64 evenly distributed distributor ports. For the test, a 20-L pulse of saturated NaCl solution was introduced through 16 distributor ports located near the sampling side. At the outlet of the fourth segment, a 50 × 20 cm plate was inserted to divert a portion of the treated water for measurement. The electrical conductivity of the effluent was recorded continuously using a WA2017SDJ conductivity logger equipped with a CDPB-03 probe (SATO TECH).

The actual HRT was calculated as the mean residence time from the conductivity breakthrough curve. The ratio of measured to designed HRT was used to evaluate the hydraulic performance of the DHS reactor before and after operational modifications (e.g., sponge sheet installation).

### Power consumption and excess sludge generation

In this study, the term “conventional process” refers to the extended‑aeration activated sludge process previously operated at the Susaki STP. Power consumption for the conventional process and for the DHS system was measured using power meters installed in the facility control panels. Daily cumulative electricity use was divided by the corresponding daily treated water volume to calculate the daily specific power consumption. In addition, the relationship between daily influent flow and specific power consumption was examined to assess energy intensity under varying flow conditions. For both systems, raw sewage pumps conveying wastewater from the manhole to the PS and screening equipment for coarse-solids removal were common components that were replaced during facility renewal to meet the current treatment capacity.

The DHS system includes a sludge-handling process in which sludge withdrawn from the PS is sent to a gravity thickener and then processed by a sludge dewatering machine to produce a dewatered cake, which is discharged off-site. All DHS effluent, together with the sludge generated during DHS operation, flows into the MBBF. The MBBF undergoes intermittent washing (approximately once every hour), generating wash water containing excess sludge. Overflows from the gravity thickener, filtrate from sludge dewatering, and MBBF backwash water are returned to the manhole pump chamber and then to the PS; therefore, these streams are not discharged outside the system. Consequently, for both the conventional process and the DHS system, the excess sludge production rate was evaluated using the monthly averages of the influent and effluent dry solids (DS) loads and the DS content of the dewatered sludge cake. The excess sludge production rate was calculated as the sum of the DS loads in the treated effluent and the dewatered sludge cake, normalized to the influent DS load.

## Results and discussion

### Performance evaluation of the DHS reactor

**Fig. 2:**
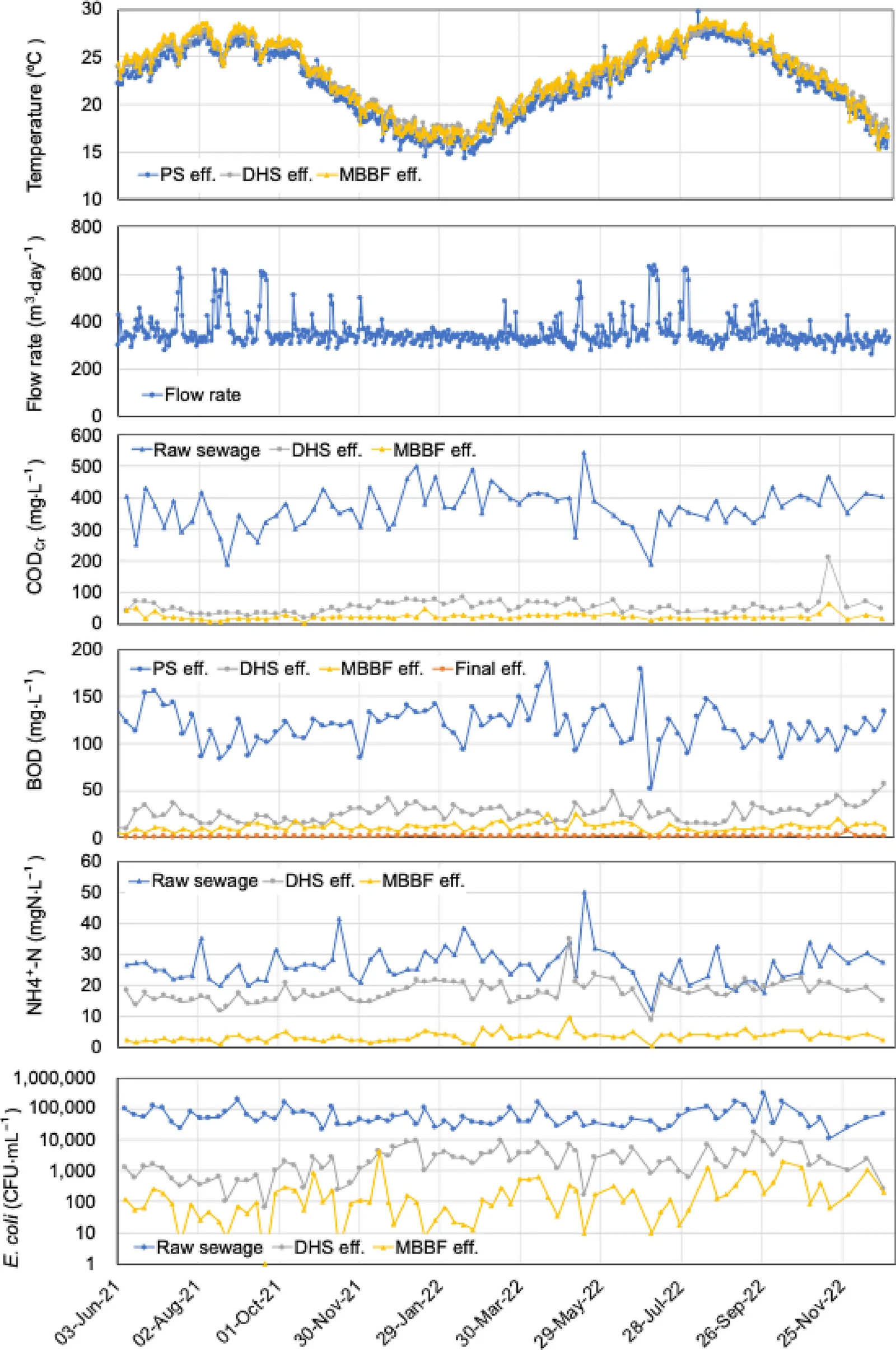
Temporal variations in water temperature, flow rate, dichromate-based chemical oxygen demand (COD_Cr_), biochemical oxygen demand (BOD), NH₄⁺–N, and *E. coli* concentrations in raw sewage and DHS effluent during Period 1 and Period 2 (June 2021–December 2022). Grab samples were collected weekly between 9:00 and 10:00 a.m

Figure 2 summarizes the long-term variations in water temperature, flow rate, and key water quality parameters for raw sewage and DHS effluents from June 2021 to December 2022. For consistency of temporal resolution, nitrogen species are graphed through December 2022 (weekly data). From March 2023 onward, monthly nitrogen measurements are summarized elsewhere (Table 1 and Section “Vertical concentration profiles and effect of sponge sheet installation”). During this period, the influent represented medium-strength domestic sewage (COD_Cr_, 368 ± 64 mg·L⁻^1^; BOD, 134 ± 35 mg·L⁻^1^; SS, 139 ± 56 mg·L⁻^1^; NH₄⁺–N, 26 ± 6 mg N·L⁻^1^; *E. coli*, 5.3 × 10^4^ CFU·mL⁻^1^; total coliforms, 1.8 × 10^5^ CFU·mL⁻^1^).

**Table 1:** Water quality parameters in raw sewage and DHS effluent during four operational periods: Period 1 (June–December 2021; normal operation), Period 1 (winter) (December 2021–March 2022; low-temperature operation), Period 2 (June–December 2022; downsized operation), and Period 3 (April–December 2023; normal operation with sponge sheet installation)

		Period 1 Jun 2021ーDec 2021				Period 1 (winter) Dec 2021ーMar 2022				Period 2 Jun 2022ーDec 2022				Period 3 Apr 2023ーDec 2023			
		Normal operation				Normal operation				Downsized operation				Normal operation + Sponge sheet			
		Raw sewage		DHS eff		Raw sewage		DHS eff		Raw sewage		DHS eff		Raw sewage		DHS eff	
BOD loading	kg·m^–3^·d^–1^	0.94	(0.38)			0.73	(0.11)			0.88	(0.17)			0.68	(0.14)		
COD_Cr_ loading	kg·m^–3^·d^–1^	2.05	(0.36)			2.19	(0.33)			2.39	(0.35)			N.A			
NH_4_^+^–N loading	kgN·m^–3^·d^–1^	0.15	(0.03)			0.15	(0.02)			0.16	(0.03)			0.14	(0.03)		
Temperature	ºC	23.1	(2.7)	24.2	(2.8)	16.9	(1.1)	18.1	(1.2)	23.5	(2.8)	24.4	(2.9)	21.4	(3.9)	23.8	(2.8)
pH	−	7.4	(0.2)	6.7	(0.1)	7.6	(0.2)	6.8	(0.1)	7.3	(0.2)	6.8	(0.1)	7.3	(0.1)	6.7	(0.1)
SS	mg·L^–1^	149	(81)	21	(7)	168	(43)	37	(16)	122	(25)	20	(6)	120	(26)	14	(6)
COD_Cr_	mg·L^–1^	341	(58)	45	(15)	401	(61)	66	(12)	361	(54)	56	(35)	N.A		N.A	
BOD	mg·L^–1^	157	(50)	22	(8)	133	(16)	29	(6)	135	(22)	29	(11)	111	(26)	22	(5)
NH_4_^+^-N	mgN·L^–1^	26	(5)	16	(2)	28	(4)	18	(3)	25	(5)	19	(3)	24	(6)	9	(2)
NO_3_^–^-N	mgN·L^–1^	0.5	(0.1)	4.9	(0.8)	0.5	(0.1)	4.6	(0.6)	0.5	(0.1)	3.4	(0.6)	N.A		6.2	(1.1)
T-N	mgN·L^–1^	34	(6)	21	(4)	37	(4)	25	(4)	34	(8)	23	(5)	32	(6)	18	(2)
*E. coli*	CFU·mL^–1^	5.90E + 04		8.20E + 02		4.40E + 04		3.30E + 03		5.20E + 04		2.50E + 03		N.A		N.A	
Coliform	CFU·mL^–1^	2.10E + 05		4.80E + 03		1.20E + 05		1.10E + 04		1.80E + 05		1.10E + 04		N.A		N.A	

Values represent means with standard deviations in parentheses. *N.A.* denotes not analyzed

Long-term monitoring of a full-scale parallel-unit downflow hanging sponge system with adaptive capacity for fluctuating wastewater flows in Japan

The parallel-unit DHS system consistently produced effluent with COD_Cr_, 55 ± 24 mg·L⁻^1^; BOD, 25 ± 8 mg·L⁻^1^; SS, 22 ± 14 mg·L⁻^1^; and NH₄⁺–N, 18 ± 3 mg N·L⁻^1^, while reducing *E. coli* and total coliform counts to 1.7 × 10^3^ and 8.6 × 10^3^ CFU·mL⁻^1^, respectively. Subsequent polishing in the anthracite-based MBBF further improved effluent quality, lowering COD_Cr_ and BOD to 24 ± 9 and 9 ± 5 mg·L⁻^1^, SS to 6 ± 6 mg·L⁻^1^, and NH₄⁺–N to 3 ± 1 mg N·L⁻^1^, with *E. coli* and total coliforms reduced to 3.0 × 10^2^ and 1.0 × 10^3^ CFU·mL⁻^1^, respectively.

Removal contributions along the PS–DHS–MBBF process indicate that DHS accounted for the majority of organics and solids removal (COD_Cr_, 85 ± 6%; BOD, 77 ± 8%; SS, 79 ± 10%) and achieved approximately 1.5 log₁₀ reduction in *E. coli* and 1.4 log₁₀ reduction in total coliforms. The MBBF provided additional polishing, particularly for ammonium removal (56 ± 11%, calculated as the PS-to-MBBF removal efficiency minus the PS-to-DHS removal efficiency), and contributed a further ~ 1.2 log₁₀ reduction in *E. coli* and 0.9 log₁₀ reduction in total coliforms. Importantly, discharge targets set at the Susaki STP (pH, 5.0–9.0; BOD < 15 mg·L⁻^1^; SS < 30 mg·L⁻^1^; coliforms < 3.0 × 10^3^ CFU·mL⁻^1^) were already met prior to final chlorination, underscoring the robustness of the PS → DHS → MBBF configuration.

This study evaluated a full-scale sewage treatment train configured as PS → DHS → MBBF, in which PS precedes the DHS reactor, and an anthracite-based MBBF provides final polishing. To the best of our knowledge, only two previous studies have reported DHS systems combined with PS as a pre-treatment step: full-scale PS + DHS in Egypt (Okubo et al. 2022) and package-type PS + DHS in Thailand (Watari et al. 2022); however, most DHS studies have focused on UASB + DHS post-treatment configurations (Tyagi et al. 2021).

Under medium-strength domestic sewage at the Susaki STP (COD_Cr,_ 368 ± 64 mg·L⁻^1^; BOD, 134 ± 35 mg·L⁻^1^), the DHS effluent averaged COD_Cr_, 55 ± 24 mg·L⁻^1^ and BOD, 25 ± 8 mg·L⁻^1^, and the subsequent MBBF polished these to COD_Cr_, 24 ± 9 mg·L⁻^1^ and BOD, 9 ± 5 mg·L⁻^1^ (June 2021–December 2022). These effluent concentrations are comparable to the performance reported by Okubo et al. (2022) (Egypt; total HRT = 3.4 h; 600 d), who achieved 82 ± 7% COD_Cr_ and 93 ± 2% BOD removal with DHS effluent of COD_Cr_, 59 ± 15 mg·L⁻^1^ and BOD, 12 ± 3 mg·L⁻^1^ across seasonal temperatures of 15–32 °C. In contrast, Watari et al. (2022) observed rapid start-up and very low DHS effluent BOD (1.1–3.9 mg·L⁻^1^) under low-strength influent (total chemical oxygen demand (COD) ≈ 42–63 mg·L⁻^1^) and short HRTs (1–4 h), illustrating the upper bound of polishing under dilute conditions. Overall, the long-term organic removal of the Susaki STP aligned with the PS-front-ended DHS at medium strength, whereas the added anthracite MBBF enabled deeper nitrification and polishing than PS + DHS alone. More broadly, the treatment performance observed in the present study was consistent with the range reported for previous DHS systems treating domestic wastewater, including UASB–DHS configurations (Tyagi et al. 2021). The present study extends these findings by providing a long-term, full-scale evaluation, along with practical operational experience, under adaptive downsizing conditions.

### Operational adjustments and performance across periods

Throughout the extended monitoring period, the Susaki system underwent four operational periods to evaluate its operational robustness and adaptability: normal operation (Period 1), winter operation under low temperature (Period 1 (winter)), partial downsizing (Period 2), and installation of a head-end sponge sheet (Period 3).

During Period 1 (June 2021–December 2021), the DHS system was operated under normal conditions with all ten units in service. Under these baseline conditions, the DHS reactor achieved stable treatment performance, producing effluent with average COD_Cr_, BOD, and NH_4_^+^–N concentrations of 45 ± 15 mg·L⁻^1^, 22 ± 8 mg·L⁻^1^, and 16 ± 2 mg·L⁻^1^, respectively (Table 1). These results were used as the reference condition for comparison with subsequent operational periods.

During Period 1 (winter) (December 2021–March 2022), wastewater temperature generally remained below 20 °C, and influent COD_Cr_ rose to 401 ± 61 mg·L⁻^1^. DHS effluent quality showed slight deterioration (COD_Cr_ 66 ± 12 mg·L⁻^1^; BOD 29 ± 6 mg·L⁻^1^), yet the subsequent MBBF consistently polished the effluent to meet discharge targets before chlorination (Fig. 2; Table 1). This reflects the typical sensitivity of organic removal to low temperatures, whereas ammonium oxidation remained comparatively stable, a trend also noted in DHS studies under cold conditions (Nomoto et al. 2018; Collings et al. 2020).

During Period 2 (June 2022–December 2022), one DHS unit was continuously operated, while nine units were intermittently rotated to simulate downsizing. Despite the reduced active sponge volume (57.6 m^3^; HRT = 3.8 h), DHS effluent remained comparable to that under normal operation (COD_Cr_ 56 ± 35 mg·L⁻^1^; BOD 29 ± 11 mg·L⁻^1^; NH₄⁺–N 19 ± 3 mg N·L⁻^1^), and the DHS–MBBF train maintained compliant final effluent. In units with a 1-week feed stoppage, a temporary decline in performance can be a concern; however, post-UASB DHS municipal sewage treatment has been reported to recover its original performance within approximately 90 min after a 10-day outage (HRT = 1.5 h) (Onodera et al. 2016). In our study, any adverse impacts were minimal, consistent with the rapid recovery of DHS biofilms under trickling conditions. These findings confirm the flexibility of the parallel-unit DHS system for capacity matching under declining inflows, which is consistent with demonstration studies highlighting DHS adaptability to flow fluctuations and its potential to reduce power demand and excess sludge generation (Maharajan et al. 2020).

Finally, during Period 3 (March–December 2023), following installation of a polyurethane sponge sheet directly below the wastewater distributor, DHS effluent NH₄⁺–N decreased to 9 ± 2 mg N·L⁻^1^, accompanied by NO₃⁻–N accumulation (6.2 ± 1.1 mg N·L⁻^1^), while BOD and TSS remained low (22 ± 5 and 14 ± 6 mg·L⁻^1^, respectively). This improvement indicates intensified nitrification and stable organics and solids polishing, consistent with previous findings that a more uniform water distribution enhances oxygen transfer and nitrification efficiency in DHS systems (Uemura et al. 2016).

**Fig. 3:**
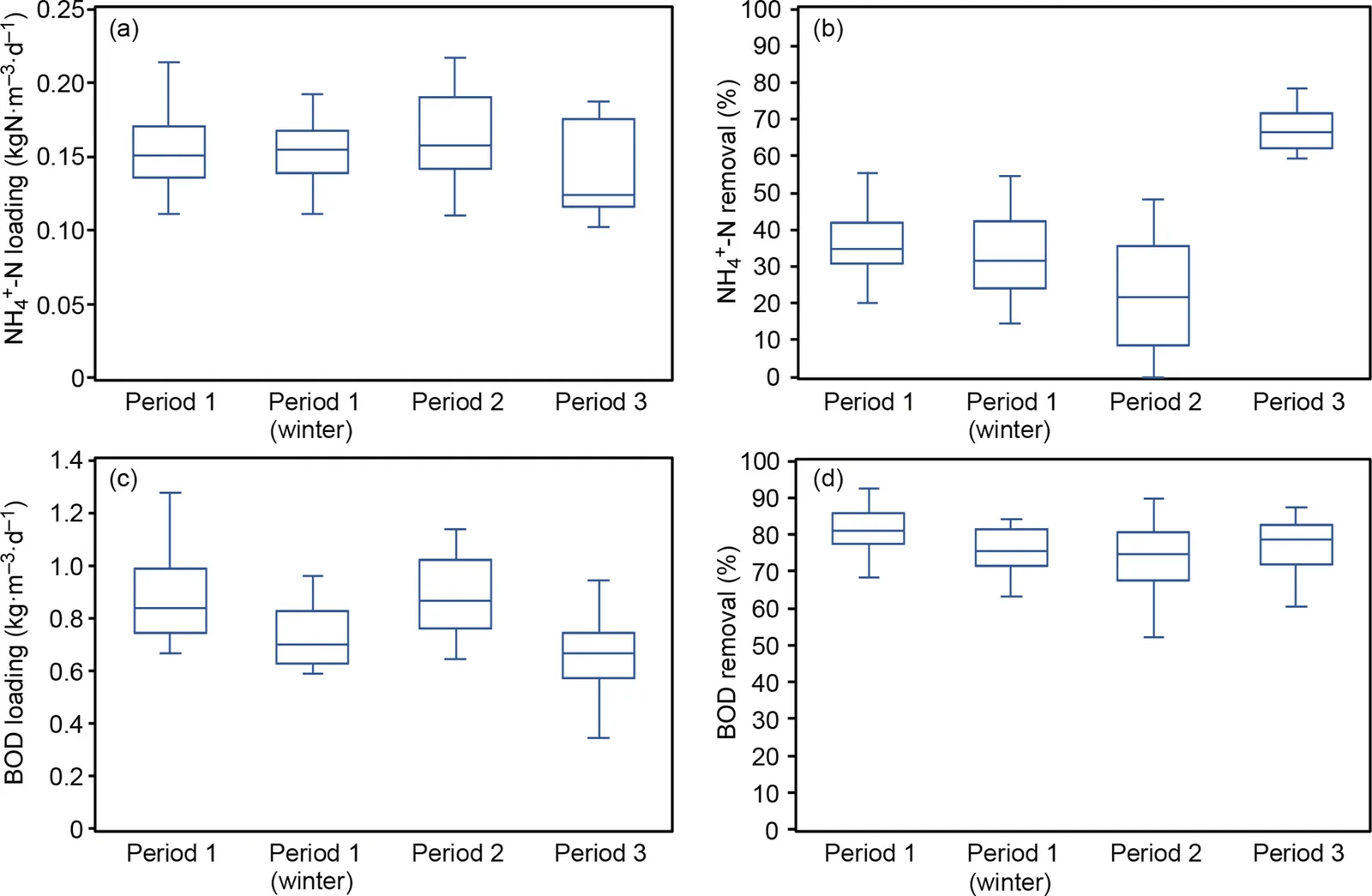
Distribution of NH_4_^+^-N loading (**a**), NH_4_^+^-N removal efficiency (**b**), BOD loading (**c**), and BOD removal efficiency (**d**) during the four operational periods. NH_4_^+^-N removal efficiency in Period 3 was significantly higher than that in the other operational periods (Games–Howell post-hoc test, *p* < 0.001)

Figure 3 compares NH₄⁺–N and BOD loading rates and removal efficiencies among the four operational periods. No significant difference in NH₄⁺–N loading was observed among the operational periods (Welch ANOVA, *p* = 0.355), indicating that the ammonia loading applied to the DHS reactor remained comparable throughout the study period. In contrast, NH₄⁺–N removal efficiency differed significantly among the operational periods (Welch ANOVA, *p* < 0.001). The highest removal efficiency was observed during Period 3 (66%), which was significantly higher than those during Period 1 (37%), Period 1 (winter) (34%), and Period 2 (22%) (Games–Howell post hoc test, *p* < 0.001). Because NH₄⁺–N loading did not significantly differ among the operational periods, the improved NH₄⁺–N removal observed during Period 3 cannot be explained solely by differences in influent loading conditions. As supporting evidence, the ammonia removal rate increased from 0.058 ± 0.021 kg·m⁻^3^·d⁻^1^ in Period 1 to 0.085 ± 0.021 kg·m⁻^3^·d⁻^1^ in Period 3. These results suggest enhanced nitrification performance in the DHS system during Period 3, potentially associated with improved wastewater distribution following the installation of the sponge sheet beneath the wastewater distributor. This interpretation is also consistent with the tracer-test results presented in Section “Vertical concentration profiles and effect of sponge sheet installation,” which indicated a modest improvement in hydraulic retention behavior after sponge sheet installation.

In contrast to NH₄⁺–N loading, BOD loading varied among the operational periods (Welch ANOVA, *p* < 0.05), ranging from 0.68 to 0.94 kg·m⁻^3^·d⁻^1^. BOD removal efficiency also differed among the operational periods (Welch ANOVA, *p* = 0.007), with average removal efficiencies ranging from 74 to 81%. However, the magnitude of variation in BOD removal was substantially smaller than that observed for NH₄⁺–N removal. These results indicate that organic matter removal remained relatively stable under different operational conditions, whereas nitrification performance was more sensitive to operational changes. As discussed in Section “Vertical concentration profiles and effect of sponge sheet installation,” sponge sheet installation was associated with increased DO and ORP levels within the DHS reactor, suggesting improved oxygen availability and, in turn, more favorable oxidation conditions for ammonia oxidation.

### Effect of feed stoppage on sludge properties

In the partial downsizing performed in April 2022, nine of the ten DHS units were operated. To examine how a 1-week feed stoppage influenced sludge properties, subsamples of sponge carriers were taken from the first segment of Unit 1 and analyzed for sludge concentration, as described in Section “Measurement of sludge concentration on sponge carriers.” The sludge concentrations measured immediately after placement in the mock DHS segment (Day 0) and on Days 3, 8, and 14 were 54.2 ± 6.6, 50.1 ± 3.5, 39.6 ± 1.7, and 38.5 ± 7.3 gVSS·L⁻^1^-sponge, respectively, indicating that approximately 30% of the sludge volume was lost by Day 8.

The decrease in sponge-attached sludge concentration during feed stoppage is attributable to the biodegradation of endogenous residues. Endogenous respiration rates tend to be higher in the upper part of the DHS reactor and lower in the lower segments, with reported values of 0.018–0.179 gO₂·gVSS⁻^1^·day⁻^1^ for a municipal UASB–DHS system (Onodera et al. 2013) and 0.08–0.33 gO₂·gVSS⁻^1^·day⁻^1^ for a DHS reactor treating low-strength domestic wastewater (Takemura et al. 2022), which are comparable to those in membrane reactors (Witzig et al. 2002) and activated sludge systems (Holm Kristensen et al. 1992). Although endogenous respiration was not measured by the oxygen uptake rate (OUR) method in this study, prior work suggests that the biodegradation rate of endogenous residue, determined by the OUR and VSS methods, yields comparable values (Ramdani et al. 2010), allowing estimation of endogenous respiration. In our case, sludge decreased from 54.2 to 39.6 gVSS·L⁻^1^-sponge over 8 d, corresponding to an apparent sludge reduction rate of 0.03–0.04 d⁻^1^. Assuming the degraded sludge exhibits a COD/VSS ratio ≈ 1.2 (Bullock et al. 1996), the endogenous respiration rate is estimated at 0.04–0.05 gO₂·gVSS⁻^1^·d⁻^1^. Considering that the sponge sample was collected from the upper (first) segment of the DHS, this estimate is slightly lower than previously reported DHS values.

Typically, measurements of endogenous residue biodegradation are performed under stirred aqueous conditions, whereas the present mock DHS segments reproduced a non-stirred trickling environment. Consequently, the degradation product concentrations were not homogeneous in the test system, which may have resulted in lower estimates than those obtained by standard stirred methods. However, these results demonstrate that sludge degradation proceeds even without mechanical mixing under DHS-like conditions.

To assess the effects on the full-scale reactor, sponge carriers were collected from Units 1, 5, 6, and 9 on May 18, 2022, across the first to fourth segments (DHS 1st–4th), and sludge concentrations were measured. Units 1 and 9 did not undergo feed stoppage; Unit 5 was sampled 2 weeks after restarting, and Unit 6 was sampled 1 week after restarting, following a 1-week feed stoppage. Across units, sludge concentration tended to be higher in the 1 st segment and lower in the 4th segment, with ranges of 32.1–54.2, 27.5–31.9, 24.5–27.5, and 24.2–29.8 gVSS·L⁻^1^-sponge for the 1 st, 2nd, 3rd, and 4th segments, respectively. Due to the substantial intrinsic variability among sponge carriers, segment-wise sludge concentrations did not consistently replicate the trends observed in the mock-segment experiment.

**Fig. 4:**
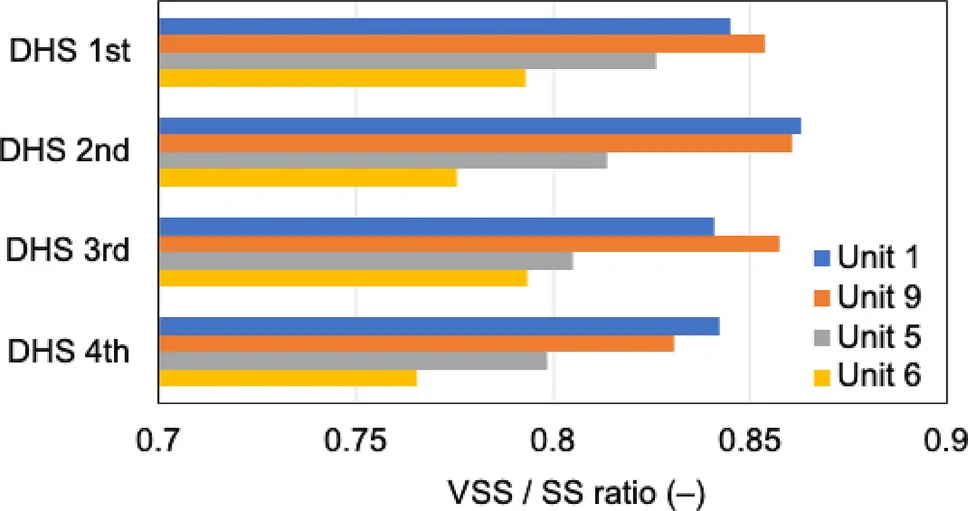
Volatile suspended solids (VSS)/total suspended solids (SS) ratio of sponge-attached sludge sampled on May 18, 2022, during Period 2 (partial downsizing operation). Units 1 and 9 were supplied continuously. Units 5 (2 weeks post-restart) and 6 (1 week post-restart) were sampled after resuming supply following a 1-week stoppage

In contrast, the VSS/SS ratio exhibited a clear segment-wise pattern (Fig. 4). The average VSS/SS ratio across the 1st–4th segments was 0.848 ± 0.009 and 0.851 ± 0.012 for Units 1 and 9 (no feed stoppage), respectively, while it was 0.811 ± 0.010 for Unit 5 (2 weeks after restart) and 0.782 ± 0.012 for Unit 6 (1 week after restart). In Unit 6, the lower VSS/SS ratio was attributed to a decrease in microbial biomass caused by sludge volume reduction during feed stoppage. The VSS/SS ratio in Unit 6 was significantly lower than those in Units 1 and 9 (Games–Howell post-hoc test, *p* < 0.001), indicating a reduction in active biomass during the feed-stoppage period. In Unit 5, partial recovery of the VSS/SS ratio suggests biomass regrowth over the 2 weeks following the restart.

### Vertical concentration profiles and effect of sponge sheet installation

**Fig. 5:**
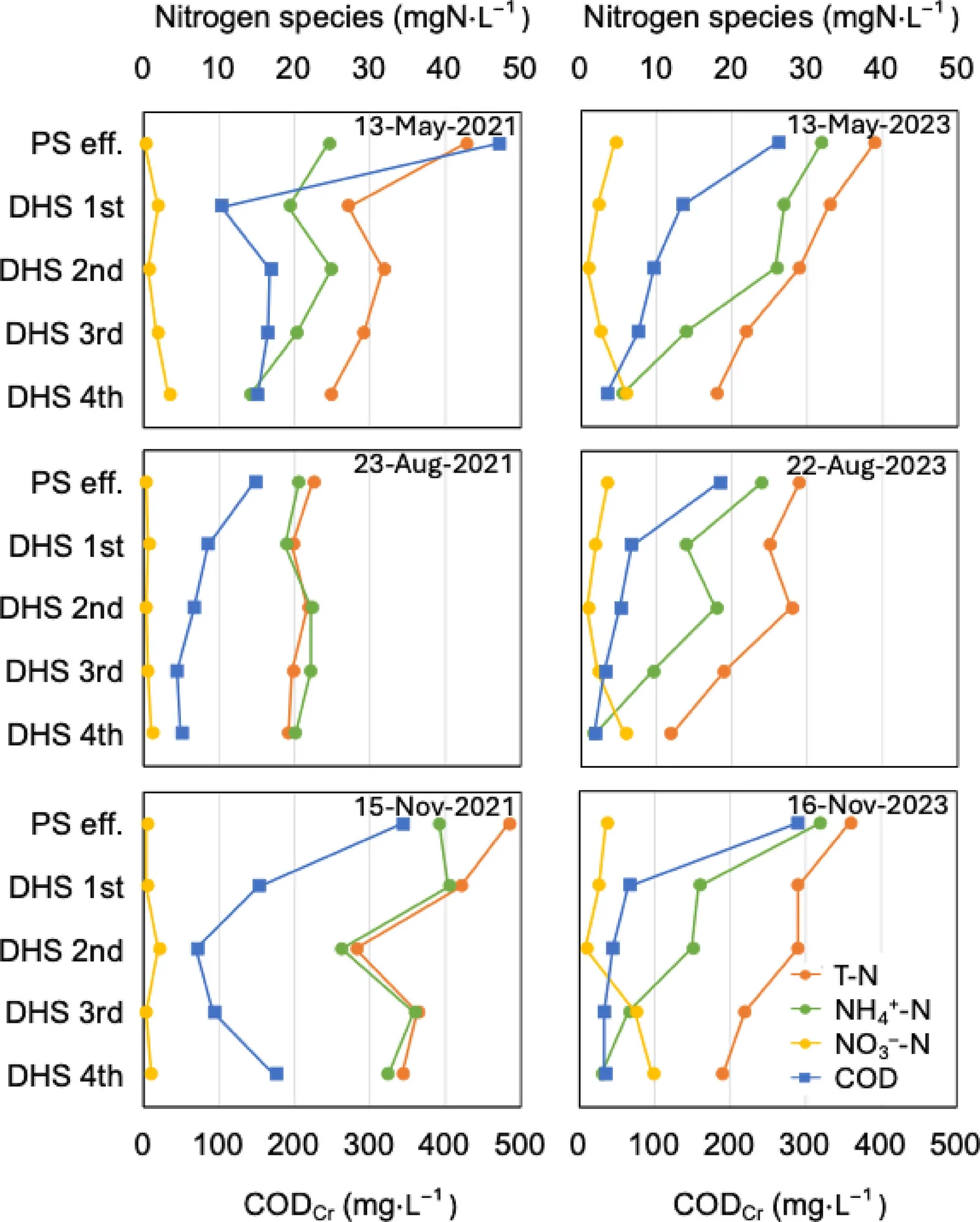
Concentration profiles of total nitrogen (TN), ammonium nitrogen (NH₄⁺-N), nitrate nitrogen (NO₃⁻-N), and COD_Cr_ along the vertical segments of the DHS reactor. The left panel shows the profiles during Period 1 (May 2021–November 2021; normal operation with ten units), whereas the right panel shows the profiles during Period 3 (May 2023–November 2023; normal operation after sponge sheet installation)

Figure 5 illustrates the vertical concentration profiles of COD_Cr_, NH₄⁺–N, NO₃⁻–N, and TN across the four reactor segments under two conditions: (i) normal operation without a sponge sheet (May–November 2021), and (ii) normal operation with a sponge sheet installed directly below the distributor (May–November 2023).

Under normal operation, COD_Cr_ and NH₄⁺–N decreased progressively from the top to the bottom segments, whereas NO₃⁻–N showed little accumulation. In addition, both COD_Cr_ and NH₄⁺–N exhibited local increases in the middle section of the DHS. This pattern likely reflects elevated concentrations of sludge containing undegraded organics, which can cause oxygen limitation due to biodegradation of endogenous residue and the associated generation of NH₄⁺–N in that zone.

After installation of the sponge sheet, COD_Cr_ and NH₄⁺–N exhibited steeper declines than during normal operation without the sheet, and clear accumulation of NO₃⁻–N was observed in the lower segments. TN also decreased with the progression of nitrification, indicating that denitrification was promoted within the depth of the reactor (Danshita et al. 2020). Although increase in NO₃⁻–N concentration associated with nitrification was observed after sponge sheet installation, TN concentration decreased from 32 ± 6 mg N·L^−1^ in the influent to 18 ± 2 mg N·L^−1^ in the DHS effluent. These results suggest that, in addition to nitrification, partial denitrification may have occurred within the DHS reactor. Previous studies have demonstrated that DHS biofilms support both nitrification and denitrification processes because oxygen gradients develop within the sponge media. The outer aerobic layer promotes ammonium oxidation, whereas oxygen-limited inner zones can support denitrification, contributing to overall nitrogen removal. Furthermore, the presence of denitrifying bacterial populations and active denitrification processes within DHS biofilms has been reported, supporting the potential for simultaneous nitrification and denitrification within the reactor (Ikeda et al. 2013; Fukui et al. 2026).

This stratification is consistent with previous DHS findings, in which organics and solids removal predominated in the upper layers, while nitrification was enhanced in the lower layers under trickling conditions (Onodera et al. 2014a, b; Nomoto et al. 2018). These changes indicate that the sponge sheet improved splash dispersion and interception at the head end, thereby enhancing oxygen transfer, intensifying nitrification, and stabilizing polishing performance throughout the reactor depth. Similar improvements in oxygen transfer efficiency and nitrification have been reported when optimizing head-end hydraulics in DHS systems (Uemura et al. 2016). To further examine the effect of sponge sheet installation on the reactor environment, DO and ORP profiles were compared before and after installation. The average DO concentration across the four DHS segments increased from 4.2–5.0 mg·L^−1^ before installation (August and November 2021) to 5.5–7.1 mg·L^−1^ after installation (August and November 2023). Likewise, the average ORP increased from −15 to 13 mV before installation to 134–141 mV after installation. These changes indicate improved oxygen availability and more favorable oxidation conditions throughout the reactor, supporting the enhanced nitrification performance observed after sponge sheet installation.

Tracer tests were conducted before and after the installation of the sponge sheet on January 18, 2022 (pre-installation) and January 17, 2024 (post-installation). The cumulative 12-h flow was 209.0 m^3^ in January 2022 and 194.5 m^3^ in January 2024. Based on the sponge volume (64 m^3^), the corresponding design HRTs were 3.67 h and 3.95 h, respectively. The measured tracer HRTs (and their ratio to design HRTs) were 3.25 h (89%) in January 2022 and 3.64 h (92%) in January 2024. This modest increase in the HRT ratio supports the improved hydraulic dispersion described above. However, the effect of the sponge sheet is likely not limited to hydraulics. Visual inspection revealed dense colonization of the sheet surface by draining flies and their larvae, as well as mites. The presence of such macrofauna in trickling filter systems is known to enhance organic matter removal (Sarai 1975); however, the extent of their contribution to treatment performance in this study requires further investigation.

Taken together, the vertical profiles and tracer data corroborate the layered functional roles within the DHS and demonstrate that sponge sheet installation significantly enhanced nitrification performance without compromising organic removal. The sponge sheet likely functioned as a temporary water-retention layer, reducing localized channeling immediately below the distributor and improving contact between wastewater, air, and attached biomass.

### Power consumption and excess sludge generation

Based on operational records for the conventional process in 2016, specific power consumption (kWh·m⁻^3^) was calculated as the daily cumulative electricity use divided by the daily treated water volume. The annual average flow in 2016 was 372 m^3^·d⁻^1^, and the main contributors to power use, listed in descending order with their specific power (share in parentheses), were: aeration blowers (0.605 kWh·m⁻^3^; 32.2%), foam water pumps (0.349 kWh·m⁻^3^; 18.6%), diffused air equipment (0.284 kWh·m⁻^3^; 15.1%), sludge pumps (0.236 kWh·m⁻^3^; 12.5%), raw sewage pumps (0.151 kWh·m⁻^3^; 8.0%), screening equipment (0.104 kWh·m⁻^3^; 5.5%), sludge rakes in the final sedimentation tank (0.074 kWh·m⁻^3^; 3.9%), and others (e.g., motorized valves; 0.078 kWh·m⁻^3^; 4.1%), resulting in a total of 1.803 kWh·m⁻^3^.

For the DHS system, using operational records in 2023, the annual average flow was 363 m^3^·d⁻^1^, and the average specific power consumption was 0.473 kWh·m⁻^3^ (including all equipment). The main contributors, again listed in descending order of specific power consumption (share), were raw sewage pumps (0.157 kWh·m⁻^3^; 33.1%), transfer pumps from PS to DHS (0.084 kWh·m⁻^3^; 17.7%), transfer pumps from DHS to MBBF (0.060 kWh·m⁻^3^; 12.6%), screening equipment (0.047 kWh·m⁻^3^; 9.9%), MBBF aeration blowers (0.045 kWh·m⁻^3^; 9.6%), DHS ventilation fans (0.030 kWh·m⁻^3^; 6.4%), PS sludge rakes (0.008 kWh·m⁻^3^; 1.7%), PS sludge withdrawal pumps (0.005 kWh·m⁻^3^; 1.0%), MBBF compressors (0.002 kWh·m⁻^3^; 0.4%), and other equipment (e.g., motorized valves; 0.036 kWh·m⁻^3^; 7.6%). In the DHS system, variations in treated flow primarily affect pump operating time; consequently, specific power consumption remains nearly constant and exhibits only a weak dependence on flow.

In the activated sludge process, aeration blowers account for the largest share of energy usage, and a minimum aeration rate must be maintained to sustain mixing and prevent diffuser clogging. Consequently, even as the treated flow decreased, the power required for the aeration blowers, foam water pumps, and diffused air equipment remained nearly unchanged, resulting in higher specific power at lower flows. Moreover, controlling DO and MLSS concentrations, which strongly affect energy use and maintainability, requires skilled wastewater operators. In the DHS system, vent openings are filtered to prevent the dispersal of drain flies and other flying insects, and ventilation fans are operated; however, their power demand is substantially lower than that of the aeration equipment in the activated sludge process. Excluding common equipment (raw sewage pumps and screening devices) shared by both systems, the process-specific power consumption for treatment in the DHS system was 0.270 kWh·m⁻^3^, approximately 83% lower than that of the conventional process previously operated at the same wastewater treatment facility treating the same wastewater stream (1.548 kWh·m⁻^3^). It should be noted that the previous facility was designed to handle a larger volume of wastewater (1800 m^3^·d⁻^1^), and the mechanical equipment was oversized relative to the actual volume treated.

Excess sludge production was assessed based on influent DS, effluent DS, and discharged DS (dewatered sludge cake). In the conventional process (2009–2016), influent DS, effluent DS, and discharged DS were 1942 kg·month⁻^1^, 69 kg·month⁻^1^, and 1130 kg·month⁻^1^, respectively, resulting in an average excess sludge production rate of 0.63. In the DHS system (2023), influent DS, effluent DS, and discharged DS were 1479 kg·month⁻^1^, 30 kg·month⁻^1^, and 822 kg·month⁻^1^, respectively, yielding an average excess sludge production rate of 0.59, corresponding to a reduction of 0.04.

The lower sludge yield in the DHS is attributed to a low food-to-microorganism ratio, high biomass concentration, and an extended sludge retention time (> 100 d). In addition, predation by macrofauna (higher organisms) within the sponge media contributed to the reduction of excess sludge, as observed in this study (Hatamoto et al. 2018).

Furthermore, the amount of dewatered sludge cake produced was 6195 kg·month⁻^1^ in the conventional process and 3145 kg·month⁻^1^ in the DHS system, representing a 50% reduction. Even accounting for the 24% lower influent DS load during DHS operation compared with the conventional process, the DHS system still achieved a substantial reduction in sludge cake production. This reduction is attributed not only to decreased production of biologically generated sludge but also to improved sludge dewaterability: a greater proportion of solids in the influent sewage settled as primary sludge, and the moisture content of the dewatered cake decreased from 82% under the conventional process to 74% in the DHS system. Moreover, reducing the amount of dewatered sludge cake is beneficial from a cost perspective, as a smaller sludge cake volume lowers the frequency and cost of sludge hauling and disposal, thereby contributing to overall operational savings.

During long-term operation, no critical DHS-specific operational failures were observed. The main maintenance requirement was periodic cleaning of distributor ports to maintain uniform wastewater distribution. Although the distributor was automatically flushed once per day, occasional clogging still occurred, and manual cleaning was conducted approximately once per year.

Several limitations of this study should be considered. The findings are based on a single full‑scale DHS system operated under site‑specific conditions; therefore, the results may not be directly transferable to other contexts. In addition, detailed analyses of microbial communities were beyond the scope of this work. Future research should evaluate parallel‑unit DHS systems at multiple full‑scale sites with different influent characteristics and climatic conditions. Further investigations integrating hydraulic, microbiological, and long‑term operational assessments would support the broader application and optimization of DHS systems.

## Conclusions

This study aimed to investigate the long‑term performance of a full‑scale, parallel‑unit DHS system and its adaptability to operational changes in direct domestic sewage treatment in a temperate region. Over 30 months of monitoring, the DHS system consistently achieved high organic removal and stable effluent quality, even during winter low-temperature operation and periods of partial downsizing. The parallel-unit configuration enabled straightforward capacity adjustment without compromising treatment efficiency, demonstrating its suitability for regions experiencing population decline and reduced inflows. Statistical analysis showed that NH₄⁺–N loading remained comparable among operational periods, whereas NH₄⁺–N removal efficiency was significantly higher after sponge sheet installation, indicating enhanced nitrification performance under similar loading conditions. The installation of a sponge sheet below the distributor improved hydraulic dispersion and oxygen transfer, thereby promoting nitrification and improving process stability. Furthermore, the DHS system exhibited substantial operational advantages over the conventional activated sludge process, including 83% lower power consumption and a considerable reduction in excess sludge generation. These findings highlight DHS as a viable, energy-efficient technology for sustainable wastewater management, particularly in small- to medium-scale facilities requiring flexible capacity and low operational costs. This study confirms that a full-scale parallel-unit DHS system can deliver robust and adaptable treatment performance for domestic sewage under fluctuating conditions and during low-temperature periods below 20˚C. In addition, the DHS system represents a socially significant solution for regions experiencing population decline, contributing to sustainable wastewater management through resilient, energy-efficient operation under demographic and economic constraints, while supporting the transition to more sustainable wastewater infrastructure management.

## Data Availability

Data are available from the corresponding author upon reasonable request.
